# Subjective visual vertical after treatment of benign paroxysmal positional vertigo^☆^

**DOI:** 10.1016/j.bjorl.2016.08.014

**Published:** 2016-09-28

**Authors:** Maristela Mian Ferreira, Maurício Malavasi Ganança, Heloisa Helena Caovilla

**Affiliations:** a Universidade Federal de São Paulo (Unifesp), Escola Paulista de Medicina (EPM), Programa de Pós-graduação em Distúrbios da Comunicação Humana, Campo Fonoaudiológico, São Paulo, SP, Brazil; b Universidade Federal de São Paulo (Unifesp), Escola Paulista de Medicina (EPM), Departamento de Otorrinolaringologia, São Paulo, SP, Brazil; c Universidade Federal de São Paulo (Unifesp), Escola Paulista de Medicina (EPM), Disciplina de Otologia e Otoneurologia, São Paulo, SP, Brazil

**Keywords:** Benign paroxysmal positional vertigo, Inner ear, Utricle, Postural balance, Vertigem posicional paroxística benigna, Orelha interna, Utrículo, Equilíbrio postural

## Abstract

**Introduction:**

Otolith function can be studied by testing the subjective visual vertical, because the tilt of the vertical line beyond the normal range is a sign of vestibular dysfunction. Benign paroxysmal positional vertigo is a disorder of one or more labyrinthine semicircular canals caused by fractions of otoliths derived from the utricular macula.

**Objective:**

To compare the subjective visual vertical with the bucket test before and immediately after the particle repositioning maneuver in patients with benign paroxysmal positional vertigo.

**Methods:**

We evaluated 20 patients. The estimated position where a fluorescent line within a bucket reached the vertical position was measured before and immediately after the particle repositioning maneuver. Data were tabulated and statistically analyzed.

**Results:**

Before repositioning maneuver, 9 patients (45.0%) had absolute values of the subjective visual vertical above the reference standard and 2 (10.0%) after the maneuver; the mean of the absolute values of the vertical deviation was significantly lower after the intervention (*p* < 0.001).

**Conclusion:**

There is a reduction of the deviations of the subjective visual vertical, evaluated by the bucket test, immediately after the particle repositioning maneuver in patients with benign paroxysmal positional vertigo.

## Resumo

**Introdução:**

A função do otólito pode ser estudada por meio de testes da vertical visual subjetiva, porque a inclinação da linha vertical além da faixa normal é um sinal de disfunção vestibular. A vertigem postural paroxística benigna é um distúrbio de um ou mais canais semicirculares labirínticos causado por frações de otólitos derivados da mácula utricular.

**Objetivo:**

Comparar a vertical visual subjetiva com o teste do balde antes e imediatamente após a manobra de reposicionamento de partículas em pacientes com vertigem posicional paroxística benigna.

**Método:**

Foram avaliados 20 pacientes. A posição estimada, onde uma linha de fluorescência dentro de um balde atingia a posição vertical, foi medida antes e imediatamente após a manobra de reposicionamento de partículas. Os dados foram tabulados e analisados estatisticamente.

**Resultados:**

Antes da manobra de reposicionamento, 9 pacientes (45,0%) apresentaram valores absolutos de vertical visual subjetiva acima da referência padrão e 2 (10,0%) depois da manobra; a média dos valores absolutos do desvio vertical foi significativamente mais baixa depois da intervenção (*p* < 0,001).

**Conclusão:**

Há uma redução dos desvios da vertical visual subjetiva, avaliada pelo teste do balde, imediatamente após a manobra de reposicionamento de partículas em pacientes com vertigem posicional paroxística benigna.

## Introduction

The perception of the vertical position depends on the integration of vestibular, proprioceptive and visual information; however, it has not yet been determined how and where in the cerebral cortex the vestibular information for spatial perception is processed.^1^ The three semicircular canals are sensitive to angular accelerations, and the utricular and saccular maculae, with their otoliths, are sensitive to linear accelerations; the cortical otolith afferents provide spatial orientation, perception of movement, and mental representation of the body in space.^2^

The benign paroxysmal positional vertigo (BPPV), recognized as a common cause of vertigo with a higher prevalence in females and in the elderly, in most cases is unilateral and idiopathic. It is characterized by episodes of sudden vertigo and/or positional nystagmus, of short duration, and may be accompanied by nausea; it occurs when there is a change of position of the head due to otolith fractionation of utricular macula, and the displacement of these particles to the semicircular canals.^3^, ^4^, ^5^, ^6^, ^7^, ^8^

The diagnosis of BPPV is based on clinical history and is established by a complaint of vertigo with visualization of nystagmus during positional maneuvers, usually paroxysmal and fatigable, and exhibiting latency. The characteristics of the positional nystagmus elicited by the Dix-Hallpike test^9^ or the test of head rotation^10^ identify the affected labyrinth and semicircular canal.^10^

There are several methods to assess the vestibular system. The test that evaluates the ability to judge whether objects are upright is called subjective vertical visual (SVV). This test can indicate impairment of spatial orientation in patients with peripheral^11^ and central^12^, ^13^ vestibular disorders, especially in acute unilateral otolith dysfunctions, indicating that the greater the deviation, the more acute or more extensive the lesion.^14^ The direction of SVV tilt is usually to the same side in unilateral peripheral (labyrinth and/or vestibular nerve) or pontomedullary (vestibular nuclei) lesions, and to the opposite side of the affected ones in unilateral pontomesencephalic lesions, and can be to the same side or the opposite side in thalamic or dentate nucleus lesions.^15^

SVV can be evaluated with different procedures, including a hemispheric dome with randomly arranged colored dots, a portable light bar, the projection of a line on a screen, and the bucket method. Since the hemispheric dome method and the “bucket method” had similar distribution of SVV values, it is concluded that the use of the “bucket method” could become part of routine clinical tests, because it is easy to apply and is inexpensive.^16^ The bucket method, applied in Brazil, showed that the highest concentration of absolute values of the vertical deviations reached a value of 3 in adults and healthy elderly individuals, regardless of gender, and did not increase with age.^17^ The limit of SVV deviation with the bucket method considered normal in adults and elderly,^17^ and in the elderly and oldest,^18^, ^19^ is similar to the limit of SVV used in other methods.^20^, ^21^

In patients with BPPV that were evaluated by different methods, SVV deviations were different^22^, ^23^, ^24^ or similar^25^ to the control group deviations. Deviations of SVV were normal,^23^ abnormal^20^, ^26^ or were near normal limits^11^ and were always or often inclined toward the affected side^11^, ^20^, ^23^ or inclined to the healthy side.^26^ With the bucket method, abnormal deviations of SVV were frequent, and often occurred to the affected side.^27^

The low cost, easy evaluation of SVV with the bucket test, and the contradictory reports in the literature about the findings in patients with BPPV motivated the interest in expanding the experience with this diagnostic method before and after treatment of this disease with maneuvers for repositioning particles.

The objective of this research is to compare the subjective vertical visual through the bucket test before and after particles repositioning maneuver in patients with benign paroxysmal positional vertigo.

## Methods

This study was approved by the Research Ethics Committee of the institution under No. 733.154/2014. All participants received information about the research and its objectives through an explanatory letter, and signed the Informed Consent before the investigation began.

In this cross-sectional study, the sample consisted of adult male or female patients diagnosed with BPPV.

The criterion for inclusion of patients was the diagnosis of BPPV, made by the otolaryngologist, based on clinical history and the presence of vertigo and positional nystagmus when undergoing Dix-Hallpike test^9^ and head rotation,^10^ to identify the impaired labyrinth (right, left or both) and the semicircular canal (posterior, anterior or lateral).

We excluded patients with inability to understand and follow simple verbal commands; severe visual impairment, or any whose vision was not compensated with the use of corrective lenses; neurological and/or mental disorders; other vestibular disorders, alcohol intake 24 h prior to evaluation; those on drugs that act on the central nervous system or the vestibular system; and those had undergone rehabilitation for body balance in the last six months.

Patients underwent, successively on the same day, history taking, investigation for vertigo and positional nystagmus, and evaluation of SVV with the bucket method^16^, ^17^ before and immediately after performing a single particle repositioning maneuver.

The investigation for vertigo and positional nystagmus in Dix-Hallpike tests^9^ and head rotation^10^ determined the clinical diagnosis of patients. The presence of nystagmus at right Dix-Hallpike test indicated right labyrinth BPPV, and at left Dix-Hallpike indicated left labyrinth BPPV. Torsional and vertical up-beat nystagmus indicated posterior canal involvement; the torsional and vertical down-beat nystagmus indicated anterior canal involvement. In the head rotation test to the right and left sides, more intense geotropic horizontal nystagmus indicated an impairment of the lateral canal on the same side, and more intense ageotropic horizontal nystagmus indicated impairment of the lateral canal on the opposite side.^10^

Depending on the identification of the impaired semicircular canal, the otolaryngologist selected Epley's particle repositioning maneuver^28^ to be performed in cases of posterior canal BPPV, and Lempert's maneuver^29^ for the lateral canal BPPV.

Binocular assessment of SVV used a bucket.^30^ At the internal bottom of the bucket, a fluorescent tape was arranged rectilinearly and perfectly aligned with the ground zero of a protractor positioned on the outside bottom of the bucket, and the true vertical relative to the Earth.

Patients, sitting with their head held upright and the visual field completely into the bucket, were instructed to look at the fluorescent line inside it. The bucket was rotated at random by the examiner clockwise and counterclockwise. Then the examiner slowly turned the bucket toward the zero degree position. Patients said “stop” when the fluorescent line reached the vertical position. Ten repetitions were performed, five clockwise and five anti-clockwise. The angular deviation from the vertical position was measured in degrees from the scale located outside the bucket. The mean of absolute values of deviations from true vertical of ten repetitions of the procedure was calculated for each patient before and after the repositioning maneuver. Values above 3, to the right or to the left, were considered abnormal.^17^, ^18^, ^19^ The direction of the slope in each case was determined by summing the values of all 10 repetitions, considering the positive or negative sign.^18^ Deviations to the right were defined as positive (bucket rotated clockwise relative to the patient), and deviations to the left negative (bucket rotated counterclockwise relative to the patient). The sum of the deviations to the right (positive) and left (negative) that were equal to zero defined a lack of prevalence in one direction over the other.

Data of the investigation were handled exclusively by the main investigator to ensure the right to confidentiality of information.

A descriptive statistical analysis to characterize the sample was performed. For quantitative variables, the minimum and maximum values were observed, and the mean values, medians and standard deviations were calculated. For qualitative variables, absolute and relative frequencies were calculated. The Wilcoxon test was used to compare SVV measurements at diagnosis and immediately after the repositioning maneuver. The power of the test was calculated, showing that the sample size was sufficient. Analyses were performed using SPSS (Statistical Package for Social Sciences) version 19; the significance level was 0.05 (5%).

## Results

SVV was evaluated in 20 patients with a diagnostic hypothesis of BPPV; 16 female and four male, aged between 51 and 89 years and a mean of 58.35 years.

The involvement of the left posterior semicircular canal was identified in 10 cases, the involvement of the right posterior semicircular canal in nine, and the right lateral semicircular canal in one.

Table 1 shows a comparison of the absolute values of SVV before and after the repositioning maneuver in 20 patients with BPPV.

**Table 1:** Absolute values and statistical data of vertical deviations and its direction before and after the repositioning maneuver in patients with benign paroxysmal positional vertigo.

Patients	Semicircular canal affected in BPPV	Before the maneuver		After the maneuver	
		Mean of absolute values in SVV (in degrees)	Prevalent direction of SVV deviation	Mean of absolute values of SVV (in degrees)	Prevalent direction of SVV deviation
1	L posterior	3.6	Right	3.1	Right
2	L posterior	3.7	Right	1.8	Right
3	L posterior	2.6	Left	1.0	Left
4	L posterior	3.1	Left	1.4	Left
5	L posterior	2.2	Right	1.2	Right
6	R posterior	2.8	Right	1.1	Left
7	R posterior	3.6	Right	2.2	Right
8	R posterior	1.3	Left	0.5	Left
9	R posterior	4.6	Right	2.8	–
10	R posterior	3.5	Right	1.7	Right
11	L posterior	2.3	Right	1.7	Right
12	R posterior	3.3	Left	2.0	–
13	L posterior	1.7	Left	0.2	–
14	R posterior	1.5	Left	1.3	Left
15	Lateral D	2.1	Right	0.5	Right
16	L posterior	2.4	Right	1.4	Right
17	L posterior	2.3	Right	1.0	Right
18	R posterior	5.3	Right	5.4	Right
19	R posterior	3.5	Left	1.4	Left
20	L posterior	1.5	Left	1.1	Left
Mean		2.8		1.6	
Median		2.7		1.4	
Minimum value		1.3		0.2	
Maximum value		5.3		5.4	
Standard deviation		1.1		1.1	
Wilcoxon test		*p* < 0.001			

R, right; L, left; –, no prevalence of direction.

Before the repositioning maneuver, nine patients (45.0%) had absolute values of SVV above the reference standard: abnormal deviations to the same side of the affected labyrinth in BPPV occurred in 5 cases (25.0%) and to the opposite side in 4 (20.0%). Eleven patients (55.0%) had deviations of the absolute values of SVV in the reference standard.

After the repositioning maneuver, two patients (10.0%) had abnormal deviations of the absolute values of SVV, in the same direction observed before the maneuver; 15 cases (75.0%) had normal deviations from the absolute values of SVV, 14 (70.0%) in the same direction, and 1 (5.0%) in the opposite direction compared to that seen before the maneuver; 3 cases (15.0%) did not show any prevalence of one direction over the other.

Comparing SVV deviations before and after the repositioning maneuver, the mean of absolute values of the vertical deviations in 20 cases of BPPV in the reference standard was significantly lower after the intervention (*p* < 0.001).

## Discussion

Vertigo triggered by head movements in BPPV is explained by the migration of calcium carbonate particles resulting from the fractionation of otoliths of the utricular macula.^3^, ^4^ The mechanical maneuvers of repositioning the debris of otolith aim to return the particles to the utricle through a sequence of movements of the head and body.^10^, ^28^

SVV tests evaluate the otolith function, especially that of the utricular macula.^14^, ^23^ There is evidence that BPPV is associated with utricular dysfunction, possibly due to degeneration of the utricular macula.^25^ Deviations of SVV were checked by means of a light bar in 87.5% of patients with acute BPPV compared to a control group.^22^ With the bucket method, the significant difference in the absolute values of SVV deviations in the comparison between healthy subjects and patients with posterior semicircular canal BPPV suggests that this test can indicate a disorder of spatial orientation in this condition.^24^

In this research, the abnormal deviation of absolute values of SVV was not necessarily to the same side of the affected labyrinth, a finding that is consistent with some research^20^, ^26^, ^27^ and not consistent with others that observed SVV deviations only to the BPPV side.^11^, ^20^, ^23^ Attempting to explain the finding of deviations to the contralateral side of BPPV, it was suggested that the area of the utricular macula affected by the loss of otoliths could be in cell fields that are sensitive to ipsilateral or contralateral cephalic inclinations; the otolith disorder could be bilateral, but would only manifest as a unilateral BPPV; or that visuovestibular integration structures developed a correction by moving SVV contralaterally to maintain harmony between visual and vestibular information.^20^

Almost half of this series of patients with BPPV (45.0%) showed abnormal absolute deviations of SVV during the acute period of the disease. The literature describes variable results in the acute phase of the disease. With the bucket method, 80.9% of the cases of BPPV showed absolute deviations of abnormal SVV,^27^ and with other methods abnormal deviations of SVV in BPPV were found in only 10.0% of the cases,^26^ and in 5 3% of cases^11^ and in 16.4% of patients diagnosed with right BPPV and in 14.2% of patients with left BPPV.^20^

Just a little more than half of the sample (55.0%) presented SVV deviation results within the standard reference in the acute phase of the disease. Such results of SVV within the limit considered normal was previously described in some patients with BPPV,^11^, ^23^, ^26^ suggesting that the otolith dysfunction of BPPV would not be very extensive in these cases.^11^, ^26^

In this study, most patients (77.8%) with abnormal absolute deviations of SVV before repositioning maneuver presented results within the reference standard after therapeutic maneuver. The reduction in SVV deviations after the repositioning maneuver in BPPV was also previously described.^23^, ^27^ The two cases that continued to show abnormal deviations of SVV perhaps had more extensive otolith involvement,^23^ stenosis or obstruction of the semicircular canal,^31^ or the maneuver failed to remove all particles,^32^ possibly requiring the repetition of repositioning procedure^33^ or multiple treatments,^34^, ^35^ for the resolution of BPPV. A greater number of maneuvers could provide greater clearance of the otoliths in the affected semicircular canal.^33^ The deviation of SVV in BPPV appears to be related to the dysfunction of the otolith organs; the elimination of semicircular canal otoliths would restore the macular structure and side effects in the utricle.^5^, ^23^

In the cases of BPPV evaluated in this study, the significant reduction of the average absolute SVV values after the repositioning maneuver suggests an immediate favorable effect of the therapeutic procedure performed, consistent with the proposition that the modification of the SVV after the therapeutic maneuver would reflect the migration of otoliths back to the utricle.^23^

This research showed that the bucket method was effective to evaluate SVV before and after the repositioning maneuver in BPPV patients. The significant reduction of the values of SVV deviations after the repositioning maneuver implies the utility of the bucket method to assess the favorable effect of this therapeutic procedure in BPPV.

## Conclusion

Evaluations using the bucket test reveal that there is a reduction of the deviations of the subjective vertical visual immediately after particle repositioning maneuver in patients with benign paroxysmal positional vertigo.

## Conflicts of interest

The authors declare no conflicts of interest.
